# ECSA: Mitigating Catastrophic Forgetting and Few-Shot Generalization in Medical Visual Question Answering

**DOI:** 10.3390/tomography11100115

**Published:** 2025-10-20

**Authors:** Qinhao Jia, Shuxian Liu, Mingliang Chen, Tianyi Li, Jing Yang

**Affiliations:** School of Computer Science and Technology, Xinjiang University, Urumqi 830017, China; 107552304069@stu.xju.edu.cn (Q.J.); 107552304033@stu.xju.edu.cn (M.C.); 107552301328@stu.xju.edu.cn (T.L.); 107552304184@stu.xju.edu.cn (J.Y.)

**Keywords:** Medical Visual Question Answering (Med-VQA), multimodal learning, visual feature extraction, deep reasoning

## Abstract

**Objective:** Medical Visual Question Answering (Med-VQA), a key technology that integrates computer vision and natural language processing to assist in clinical diagnosis, possesses significant potential for enhancing diagnostic efficiency and accuracy. However, its development is constrained by two major bottlenecks: weak few-shot generalization capability stemming from the scarcity of high-quality annotated data and the problem of catastrophic forgetting when continually learning new knowledge. Existing research has largely addressed these two challenges in isolation, lacking a unified framework. **Methods:** To bridge this gap, this paper proposes a novel Evolvable Clinical-Semantic Alignment (ECSA) framework, designed to synergistically solve these two challenges within a single architecture. ECSA is built upon powerful pre-trained vision (BiomedCLIP) and language (Flan-T5) models, with two innovative modules at its core. First, we design a Clinical-Semantic Disambiguation Module (CSDM), which employs a novel debiased hard negative mining strategy for contrastive learning. This enables the precise discrimination of “hard negatives” that are visually similar but clinically distinct, thereby significantly enhancing the model’s representation ability in few-shot and long-tail scenarios. Second, we introduce a Prompt-based Knowledge Consolidation Module (PKC), which acts as a rehearsal-free non-parametric knowledge store. It consolidates historical knowledge by dynamically accumulating and retrieving task-specific “soft prompts,” thus effectively circumventing catastrophic forgetting without relying on past data. **Results:** Extensive experimental results on four public benchmark datasets, VQA-RAD, SLAKE, PathVQA, and VQA-Med-2019, demonstrate ECSA’s state-of-the-art or highly competitive performance. Specifically, ECSA achieves excellent overall accuracies of 80.15% on VQA-RAD and 85.10% on SLAKE, while also showing strong generalization with 64.57% on PathVQA and 82.23% on VQA-Med-2019. More critically, in continual learning scenarios, the framework achieves a low forgetting rate of just 13.50%, showcasing its significant advantages in knowledge retention. **Conclusions:** These findings validate the framework’s substantial potential for building robust and evolvable clinical decision support systems.

## 1. Introduction

Medical Visual Question Answering is a key multimodal learning task that integrates advanced computer vision and natural language processing techniques, aiming to build intelligent systems capable of understanding details in medical images and generating precise answers to clinical questions, thereby playing an important auxiliary role in clinical diagnosis and decision-making [1,2]. Unlike general-domain Visual Question Answering (VQA), Med-VQA faces more severe challenges [3]. Its models not only require excellent visual feature extraction capabilities to identify subtle pathological markers but also must possess strong language understanding abilities to parse specialized medical terminology and achieve deep reasoning and fusion of information from both modalities in high-risk clinical environments [1,4]. As highlighted in the review by Dong et al., the ultimate goal of Med-VQA systems is to alleviate the burden on medical professionals and reduce the diagnostic error rates, and its inherent complexity requires the development of specialized architectures that surpass general-purpose models [1].

However, the development and deployment of Med-VQA systems are dually constrained by the inherent characteristics of medical data, giving rise to two core challenges: few-shot generalization and continual learning [5]. First, the cost of annotating high-quality medical images is extremely high, requiring significant time and effort from clinical experts, which leads to a scarcity of large-scale high-quality labeled datasets [6]. Chen et al. have pointed out that acquiring a small number of annotations is feasible in practical clinical settings, making this problem naturally suited to the paradigm of few-shot learning [7,8]. Meanwhile, the natural distribution of diseases exhibits a typical long-tail characteristic, where imaging data for rare diseases are inherently scarce [9,10]. These dual factors make it difficult for models to learn robust feature representations under few-shot conditions, resulting in poor generalization to unseen or rare cases [11]. Second, the clinical environment is dynamically evolving, with new diseases, diagnostic criteria, and imaging technologies constantly emerging. This requires AI models to possess the capability for continual or lifelong learning—that is, to continuously acquire knowledge from new tasks without accessing past data [12,13]. However, as demonstrated by Verma et al. in their research on medical image classification tasks, traditional models encounter catastrophic forgetting during the sequential learning process—where memory of previously learned knowledge catastrophically degrades while learning new information [12,14]. Therefore, any Med-VQA framework intended for clinical application must simultaneously overcome the dual challenges of weak few-shot generalization and catastrophic forgetting in continual learning.

To address the aforementioned challenges, existing methods have explored various avenues, but they still exhibit significant limitations. Early Med-VQA research primarily employed discriminative models, which constrain answers to a predefined closed set; this approach cannot handle open-ended questions and struggles to adapt to the introduction of new knowledge [1,15]. In recent years, the research focus has shifted towards generative models. For example, the Multimodal Prompt Retrieval (MPR) model proposed by Ossowski and Hu enhances the model’s domain adaptation capability in few-shot scenarios by retrieving similar image–text pairs to construct prompts [16]. However, such methods rely on a static pre-constructed retrieval library and lack a mechanism for dynamically updating the knowledge base during continual learning. Concurrently, the rise of large-scale pre-trained foundation models has introduced a new paradigm to Med-VQA. On one hand, Vision-Language Models (VLMs) like MaCo have acquired powerful zero-shot visual understanding capabilities through contrastive learning on massive radiology image–text pairs [17]. On the other hand, Large Medical Language Models (LLMs) such as GatorTron have mastered a rich body of medical knowledge through pre-training on vast clinical texts [2]. Although these models perform exceptionally well in their respective modalities, when directly applied to continual learning scenarios, the method of updating model parameters via fine-tuning is highly prone to disrupting the general knowledge embedded in the pre-trained weights, thereby triggering catastrophic forgetting [18]. In summary, current approaches either cannot generate free-text answers, lack a mechanism for dynamic knowledge updates, or perform poorly in continual learning, failing to provide a unified solution that simultaneously addresses both few-shot generalization and catastrophic forgetting.

To bridge this research gap, this paper proposes a novel Evolvable Clinical-Semantic Alignment (ECSA) framework, which skillfully integrates the core ideas of few-shot learning and continual learning, aiming to achieve efficient knowledge transfer, retention, and accumulation. The main contributions of this paper are as follows:

1. We introduce the novel Evolvable Clinical-Semantic Alignment (ECSA) framework, designed to synergistically address the dual challenges of few-shot generalization and rehearsal-free continual learning within a unified architecture for the Med-VQA task. ECSA is built upon powerful pre-trained vision (BiomedCLIP) and language (Flan-T5) foundation models and achieves continual knowledge accumulation through a parameter-efficient prompt-based learning strategy.
2. We design the Clinical-Semantic Disambiguation Module (CSDM), which achieves deep alignment of multi-scale visual features and text embeddings via a cross-attention mechanism. This hierarchical fusion strategy not only enables the model to simultaneously capture both the global context and local lesion details of an image, but more critically, it enhances the model’s ability to distinguish hard negative samples—that is, distractors that are visually highly similar to the target but belong to a different class. By precisely filtering these confusing negative samples, CSDM significantly improves the model’s representation and recognition capabilities on few-shot and long-tail rare cases.
3. We introduce the Prompt-based Knowledge Consolidation Module (PKC), which is a dynamically expandable memory store for storing task-specific “soft prompts” acquired through learning. When learning a new task, this module only adds and trains new prompts relevant to the new task while freezing the old ones, thereby effectively preserving historical knowledge without storing any past patient data and fundamentally mitigating the problem of catastrophic forgetting.

We conducted extensive experiments on four widely-used medical VQA benchmark datasets: VQA-RAD [19], SLAKE [20], PathVQA [21], and VQA-Med-2019 [22]. The results show that our proposed ECSA framework achieves state-of-the-art or competitive performance across all datasets. This fully validates its substantial potential for building robust and evolvable clinical decision support systems.

## 2. Related Work

Answering (Med-VQA) primarily revolves around two core challenges: few-shot generalization and continual learning. The former requires models to be capable of learning about rare conditions from a few samples, while the latter requires models to avoid forgetting old knowledge as they continuously learn new information. Although existing research has made progress in various directions, these problems are often addressed in isolation. This section aims to systematically review the related literature, first by analyzing the fundamental challenges of Med-VQA and the problem of “catastrophic forgetting” in continual learning; second, by delving into the various prompt-based learning strategies developed to counter forgetting; subsequently, by highlighting the Retrieval-Augmented Generation (RAG) paradigm, which has emerged to ensure clinical safety; and finally, by integrating these research threads, to identify the existing gaps in current research, thereby demonstrating the novelty and necessity of the framework proposed in this paper.

### 2.1. Fundamental Challenges and Continual Learning in Medical VQA

Visual Question Answering models primarily follow two design paradigms: discriminative or generative. Discriminative approaches treat VQA as a classification task, selecting an output from a predefined closed set of answers [16,23]. Although this approach provides stable outputs, it suffers from poor generalization in data-scarce medical scenarios and cannot handle the open-ended questions common in clinical practice. In contrast, generative approaches based on large language models can generate answers in free-text form, offering greater flexibility, but they also introduce the severe risk of “Factual Hallucination”—meaning the model might fabricate pathological features that are not present in the image, which is unacceptable in clinical applications [24].

At the same time, the continual development of medical knowledge requires that Med-VQA systems must possess the capability for continual learning, i.e., to continuously learn from new tasks without accessing past data. However, deep neural networks universally face the core obstacle of “catastrophic forgetting” in continual learning. When a model is trained on a new task, the parameters adjusted to adapt to the new data disrupt the previously learned knowledge, leading to a sharp decline in performance on old tasks [25,26,27].

This issue is particularly pronounced in VQA tasks. In their work VQACL, Zhang et al. (CVPR 2023) first established a formal benchmark for continual learning in VQA and demonstrated through evaluation that standard CL methods designed for single modalities generally suffer from severe catastrophic forgetting on VQA tasks [28]. The root cause lies in the inherent “dual-modality forgetting” characteristic of VQA. Recent research has defined this as a “dual form of catastrophic forgetting,” where a model not only forgets its previously learned visual understanding capabilities, but its instruction-following and language reasoning abilities also degrade when learning a new task. This indicates that an effective continual learning strategy for VQA must be able to simultaneously manage and protect knowledge from both the visual and language channels [29,30].

### 2.2. Prompt-Based Forgetting Mitigation Strategies

To tackle the complex problem of catastrophic forgetting in VQA, prompt-based learning has emerged as a fruitful research direction. This approach involves introducing a small number of learnable parameters (i.e., “prompts”) into the model to guide the behavior of a large-scale pre-trained model, thereby achieving effective adaptation to new tasks without significantly altering the main model parameters [31].

Research in this area has generally evolved along a path from simple to complex and from abstract to modality-aware. One of the early strategies was to use prompts as a medium for knowledge replay. For instance, “Symbolic Replay” proposed by Lei et al. (AAAI 2023) uses scene graphs as a highly abstract symbolic prompt to generate pseudo-samples for consolidating old reasoning mechanisms when learning new tasks [32]. However, such methods are highly dependent on expensive structured semantic information, making them difficult to apply at scale in the medical imaging domain.

To overcome this limitation, subsequent research has shifted towards the learning and management of prompts themselves, with the core idea being to learn different dedicated prompts for different tasks and store them in a shared “Prompt Pool”. The L2P (Learning to Prompt) framework proposed by Wang et al. (CVPR 2022) is a foundational work in this paradigm [33]. During inference, the L2P framework can dynamically retrieve and activate the most relevant prompts from the pool based on the current input to guide decision-making, and it does not require knowledge of the task identity at test time, which allows it to be applied in more challenging scenarios. Building on L2P, subsequent works such as DualPrompt [34] and CPrompt [35] have further optimized this mechanism.

Despite the success of the prompt pool mechanism, when applied to VQA, they still have not fully resolved the “dual-modality forgetting” problem. To tackle this challenge head-on, Qian et al. (ICCV 2023) proposed the TRIPLET model, with its core idea being to “decouple before interact” [36]. Instead of using a unified prompt, this method designs and learns decoupled prompts for the visual and language modalities separately, which are then fused through a carefully designed prompt interaction strategy. This design explicitly responds to the dual-modality nature of the VQA task, allowing the model to manage and protect knowledge from different modalities in a more fine-grained manner [37]. However, how to efficiently manage a dynamically expanding prompt library and preserve task knowledge without storing any historical data (i.e., rehearsal-free) remains an open challenge that requires further investigation.

### 2.3. Retrieval-Augmented Generation for Factual Grounding

The aforementioned “Factual Hallucination” is a core bottleneck limiting the clinical application prospects of generative Med-VQA models. To fundamentally address this issue, the Retrieval-Augmented Generation (RAG) paradigm emerged [38]. The core idea of RAG is to combine the reasoning and generation capabilities of large language models with the accuracy of an external trustworthy knowledge base [39]. It transforms the model from an omniscient “knowledge base” into an efficient “information processor”: before generating an answer, the model first retrieves relevant evidence from a trustworthy external knowledge source (such as medical literature, case files, etc.) based on the current question and then uses this evidence as context to guide the generation of a factually-grounded answer.

In the general VQA domain, RAG has been proven to be an effective means of handling knowledge-intensive problems. For example, FLMR, proposed by Gui et al. (NeurIPS 2023), introduces a “fine-grained, late-interaction” retrieval mechanism that allows the model to capture more subtle semantic associations, significantly improving the quality of retrieved documents and thus providing more reliable evidence for answer generation [40]. This paradigm is particularly crucial for Med-VQA and has evolved into various domain-adaptive variants. Some research efforts enhance factual accuracy by introducing domain-aware multimodal retrieval [41]. For instance, the MMed-RAG (ICLR 2025) system can perform targeted retrieval based on the medical sub-domain of the question (e.g., radiology, pathology) and incorporates preference fine-tuning strategies to teach the model how to better utilize the retrieved context [42]. Another line of thought, inspired by the clinical practice of referencing similar cases, has led to the proposal of Visual RAG (V-RAG). These methods not only retrieve textual knowledge but also retrieve visually similar case images and their reports from a database, providing the model with richer multimodal evidence, thereby effectively reducing hallucinations when dealing with rare or atypical cases [43]. Furthermore, to address complex problems requiring multi-step reasoning, the Agentic RAG framework has been proposed. Works like OmniSearch introduce AI agents capable of planning, which can decompose a complex problem into a chain of sub-problems and dynamically plan retrieval actions for each sub-problem, simulating the reasoning process of an expert [44].

### 2.4. Knowledge Alignment and Explainability

The ultimate goal of building advanced Med-VQA systems is not only to improve accuracy but also, more importantly, to ensure the model’s trustworthiness and explainability, such that its internal “decision logic” can be “aligned” with the medical knowledge systems of human experts.

Aligning the representation space of general-purpose models with the specific knowledge of the medical domain is crucial. Due to the unique characteristics of medical images and text, general-purpose models encounter domain gap issues when applied directly. To this end, researchers have proposed various domain-specific alignment strategies. For example, CP-CLIP, proposed by Yu et al. (MICCAI 2024), is inspired by the “core–periphery” network structure in brain science and designs a novel alignment mechanism to align medical image and clinical text features into a unified semantic space [45]. MMCAP, proposed by Yan et al. (ACL 2024), effectively integrates structured medical prior knowledge into the model by constructing a multimodal concept alignment pre-training that incorporates a medical knowledge graph [46]. However, a key challenge remains: how to enhance the model’s ability to distinguish “hard negative samples” that are visually highly similar but belong to different classes. This is crucial for accurate diagnosis in few-shot and long-tail rare case scenarios; however, existing alignment methods have paid insufficient attention to this issue [47,48].

Secondly, prompt learning and RAG also provide new avenues for enhancing model explainability. Prompt learning makes the model’s behavior more controllable and transparent by introducing explicit instructions. For example, the XCoOp framework proposed by Bie et al. (MICCAI 2024) achieves an explainable diagnostic model by aligning image features with diagnostic text using clinical concept-driven prompts [49]. RAG, on the other hand, provides users with a direct verification channel by explicitly displaying the external evidence it relies on, thereby greatly enhancing the model’s explainability and trustworthiness.

In summary, the existing research offers various effective pathways for improving Med-VQA performance, but most address specific challenges in isolation. For instance, prompt learning is primarily used to address the forgetting problem in continual learning, while RAG focuses on solving the hallucination problem in generative models. Although knowledge alignment techniques have advanced, the ability to discriminate hard negative samples in few-shot scenarios still needs to be strengthened. Therefore, the field currently lacks a solution that can, within a unified framework, synergistically solve the two core challenges of few-shot generalization and rehearsal-free continual learning. This study aims to fill this gap with the Evolvable Clinical-Semantic Alignment framework, which innovatively designs the Clinical-Semantic Disambiguation Module to enhance the discrimination of hard negative samples and the Prompt-based Knowledge Consolidation Module to achieve efficient rehearsal-free knowledge accumulation, thereby building a more robust, adaptive, and trustworthy Med-VQA system [50].

## 3. Methods

**Figure 1:**
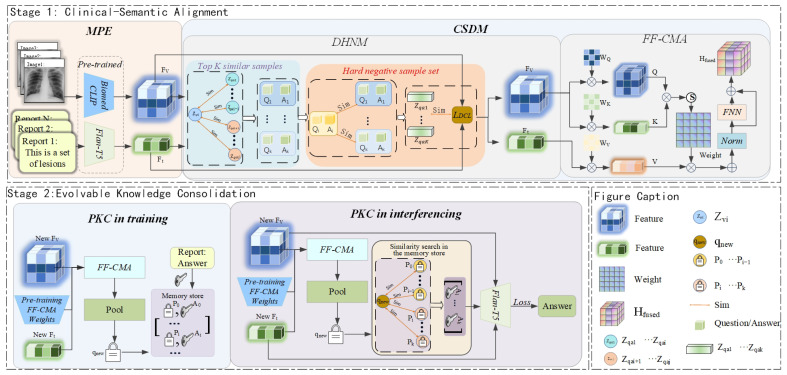
Overall schematic of the Evolvable Clinical-Semantic Alignment (ECSA) framework. The framework consists of two core stages. Stage 1: Clinical-Semantic Alignment, responsible for constructing high-quality cross-modal representations. The Multimodal Prompt Encoding (MPE) module first encodes the input, after which the Clinical-Semantic Disambiguation Module (CSDM), through a Feature Fusion Cross-Modal Attention (FF-CMA) mechanism and a Debiased Hard Negative Mining (DHNM) strategy, produces robustly aligned features for cases that are visually similar but clinically distinct. Stage 2: Evolvable Knowledge Consolidation, responsible for the model’s continual learning and knowledge generation. The Prompt-based Knowledge Consolidation (PKC) module utilizes a dynamic non-parametric memory store and employs a Retrieval-Augmented Generation mechanism during inference to provide traceable knowledge support for the final answer generation.

This section details our innovative Evolvable Clinical-Semantic Alignment (ECSA) framework, designed to address the dual challenges of precise clinical discrimination and verifiable knowledge generation in Med-VQA. The framework’s architecture is organized into two core stages, each directly reflecting a key aspect of its name. The first stage, Clinical-Semantic Alignment, establishes the model’s foundational understanding by processing input via Multimodal Prompt Encoding and then leveraging our Clinical-Semantic Disambiguation Module (CSDM) with its novel debiased hard negative mining strategy to construct a highly discriminative embedding space. Building on this, the second stage, Evolvable Knowledge Consolidation, endows the framework with its crucial adaptive capability through the Prompt-based Knowledge Consolidation Module (PKC), which functions as a Retrieval-Augmented Generation mechanism to facilitate continual learning and ensure the knowledge base remains current without catastrophic forgetting. Following this two-stage exposition, we will describe the overall training and inference process. The complete framework is illustrated in Figure 1.

### 3.1. Multimodal Prompt Encoding (MPE)

To provide an information-rich and structurally uniform input for the subsequent alignment, retrieval, and generation tasks, this framework jointly encodes a complete VQA triplet (image V, question Q, answer A) as a holistic “multimodal prompt”. This approach ensures that the model, when processing the input, can fully leverage the visual information from the image as well as the complete semantic context embedded in the question–answer pair, thereby laying the foundation for deep semantic understanding and alignment.

#### 3.1.1. Vision Feature Extraction

In medical image analysis, a model’s performance is highly dependent on its ability to extract features with clinical diagnostic value from images. To address this issue, we select the Vision Transformer (ViT) from BiomedCLIP, pre-trained on the large-scale biomedical image–text pair dataset PMC-15M, as the image encoder [51]. This model has demonstrated excellent performance in multiple biomedical vision-language tasks, and its domain-specific pre-training endows it with a stronger capability for medical concept representation.

To preserve local pathological details that are crucial for diagnosis, we do not use a single global feature vector. Instead, we process the input image *V* through the ViT backbone of the BiomedCLIP encoder. We extract the sequence of patch embeddings from its penultimate layer, a practice that strikes a good balance between spatial detail and semantic abstraction. This process can be formally defined as

(1) $$F_{v}=\mathrm{BiomedCLIP}\left(V\right),$$

where $F_{v}\in R^{N_{v}\times d_{v}}$ represents the sequence of visual features, where $N_{v}$ is the length of tokens, and $d_{v}$ is the feature dimension of each token. Pseudocode for the architectural details of the BiomedCLIP encoder has been provided in the Appendix A.

#### 3.1.2. Textual Context Encoding

The Med-VQA task requires the model not only to understand the question but also to learn by associating it with the corresponding ground-truth answer *A*. Therefore, we concatenate the question *Q* with the corresponding ground-truth answer *A* and prepend a task-specific instruction $I_{task}$ to form a complete text input $T=[I_{task};Q;A]$. This combined text is then tokenized and fed into the encoder of Flan-T5 [52] to generate a sequence of context-aware token embeddings. This process can be formulated as

(2) $$F_{t}=\text{Flan-T}5\left(T\right),$$

where $F_{t}\in R^{N_{t}\times d_{t}}$ is the embedding sequence of text tokens, where $N_{t}$ is the length of the token sequence, and $d_{t}$ is its feature dimension. Pseudocode for the architectural details of the Flan-T5 encoder has been provided in the Appendix A.

### 3.2. Clinical-Semantic Disambiguation Module (CSDM)

The CSDM module is central to achieving high-precision diagnostic reasoning within our framework. Its primary objective is to construct a shared highly discriminative multimodal embedding space that enables the model to precisely distinguish pathological states that are visually similar but clinically distinct. To achieve this goal, the CSDM module employs a refined training strategy. This strategy first confronts the unique “False Negative Trap” in medical contrastive learning through our proposed Debiased Hard Negative Mining strategy; subsequently, it leverages a feature fusion mechanism based on cross-modal attention to provide deeply aligned information-rich image–text representations for this mining strategy. Finally, the entire module is directly driven and optimized by a unified contrastive loss function.

#### 3.2.1. Debiased Hard Negative Mining for Robust Representation (DHNM)

A fundamental challenge in medical image analysis lies in the high degree of visual similarity between samples of different pathological states—that is, samples that are very close to the anchor in the embedding space but have different labels. This makes it difficult for standard models to learn discriminative features. Contrastive Learning is a powerful paradigm for addressing such tasks, with the core objective of learning an embedding space where semantically similar samples are pulled closer and semantically dissimilar ones are pushed farther apart. However, in the medical domain, this contrastive learning strategy heavily relies on hard negative samples during training, resulting in a fatal flaw, which this section terms the “False Negative Trap”. The root cause is that many different diseases or pathological states can be highly similar visually. For example, in a batch processing lung cancer images, the anchor sample $\left(V_{i},Q_{i},A_{i}\right)$ is ‘lung adenocarcinoma,’ while another sample in the batch, $\left(V_{j},Q_{j},A_{j}\right)$, is ‘lung squamous cell carcinoma.’ Due to the high visual similarity of these two types of lung cancer on imaging, a model that relies solely on cross-modal embedding similarity is highly likely to incorrectly identify the text pair $\left(Q_{j},A_{j}\right)$ as a ‘hard negative sample’ for the image $V_{i}$. When the model is trained to push these two samples apart, it is actually learning to erase the subtle yet critical diagnostic features that distinguish these two similar cancers, which severely undermines its clinical diagnostic capability.

**Figure 2:**
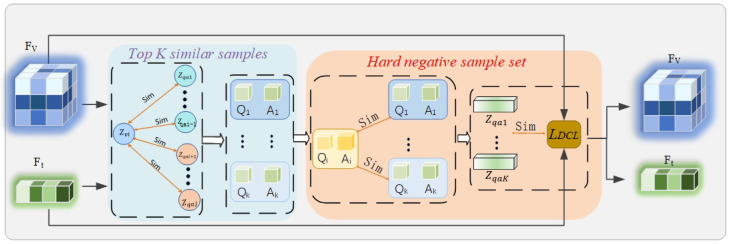
Flowchart of the Debiased Hard Negative Mining for Robust Representation (DHNM) module. For an anchor image feature $F_{v}$ within a batch, visual similarity is first computed against all textual features $F_{qa}$ in the batch, and the Top-K most similar texts are selected as a candidate set. Subsequently, an independent semantic encoder (Semantic Referee) is used to calculate the semantic similarity between these candidate texts and the anchor text. Only those candidate samples with high visual similarity but low semantic similarity are ultimately confirmed as ‘true’ hard negative samples for the subsequent contrastive learning loss calculation.

To address this issue, we propose a novel Debiased Hard Negative Mining for Robust Representation (DHNM). The overall framework of DHNM is shown in Figure 2. First, we map the high-dimensional feature sequences to the contrastive learning embedding space via a pooling operation (denoted as Pool(·)) and a projection head (a small MLP, denoted as ϕ) to obtain normalized global representations $z_{v}=\phi_{v}\left(\mathrm{Pool}\left(F_{v}\right)\right)$ and $z_{qa}=\phi_{qa}\left(\mathrm{Pool}\left(F_{t}\right)\right)$. For an anchor *i* in a batch of size *B*, we compute the cosine similarity between its image representation $z_{v_{i}}$ and all in-batch text representations $z_{qa_{j}}$ (where j≠i), and select the indices of the Top-K most similar samples to form a candidate set:

(3) $$J_{\mathrm{cand},i}=\arg\underset{j\in \{1,\ldots ,B\},j\neq i}{\text{top-k}}\left(\mathrm{sim}\left(z_{v_{i}},z_{qa_{j}}\right)\right).$$

The corresponding hard negative sample set is $N_{\mathrm{cand},i}=\left\{\left(Q_{j},A_{j}\right)|j\in J_{\mathrm{cand},i}\right\}$. Next, we perform semantic filtering on the candidate set $N_{\mathrm{cand},i}$. We utilize the PubMedBERT text encoder integrated within the BiomedCLIP model as a “semantic referee” $E_{\mathrm{sem}}$. For each candidate negative sample $\left(Q_{j},A_{j}\right)$ in $N_{\mathrm{cand},i}$, we compute its semantic similarity with the anchor text $\left(Q_{i},A_{i}\right)$. To avoid the “False Negative Trap”, we discard candidate samples that are semantically too similar to the anchor, retaining only the semantically irrelevant ones as the final hard negative sample set $N_{\mathrm{hard},i}$. The filtering condition is controlled by a preset threshold θ, a hyperparameter (set to 0.85 in this study):

(4) $$N_{\mathrm{hard},i}=\left\{\left(Q_{j},A_{j}\right)|j\in J_{\mathrm{cand},i}\;\mathrm{and}\;\mathrm{sim}\;\left(E_{\mathrm{sem}}\left(Q_{i},A_{i}\right),\;E_{\mathrm{sem}}\left(Q_{j},A_{j}\right)\right)\leq \theta \right\},$$

where sim(·,·) represents the cosine similarity. This process ensures that only those samples that are visually confusing, but are indeed clinically-semantically irrelevant, are used for the subsequent contrastive learning.

#### 3.2.2. Unified Debiased Contrastive Loss

To directly utilize the high-quality negative samples mined in the previous step to optimize the CSDM module, we adopt a unified optimized InfoNCE loss function $L_{\mathrm{DCL}}$. This loss frames the contrastive learning task as a multi-class classification problem. For a given anchor image $z_{v_{i}}$, its objective is to distinguish the corresponding positive text sample $z_{qa_{i}}$ from all the true hard negative samples (from $N_{\mathrm{hard},i}$) within a set. Its formal definition is as follows:

(5) $$L_{DCL}=-\log\left(\frac{\exp(\mathrm{sim}\left(z_{v_{i}},z_{qa_{i}}\right)/\tau )}{\exp\left(\mathrm{sim}\left(z_{v_{i}},z_{qa_{i}}\right)/\tau \right)+\sum_{\left(Q_{j},A_{j}\right)\in N_{\mathrm{hard},i}}\exp\left(\mathrm{sim}\left(z_{v_{i}},z_{qa_{j}}\right)/\tau \right)}\right),$$

where $z_{qa_{j}}$ is the embedding representation corresponding to the hard negative sample $\left(Q_{k},A_{k}\right)$, and τ is the temperature hyperparameter. The InfoNCE loss is itself a hardness-aware loss function: the temperature parameter τ naturally controls the degree of focus on hard negative samples.

The objective of this loss function is to increase $\exp(\mathrm{sim}(z_{v_{i}},z_{qa_{i}})$ to pull $z_{v_{i}}$ and $z_{qa_{i}}$ closer in similarity, while simultaneously decreasing $\exp(\mathrm{sim}(z_{v_{i}},z_{qa_{j}})$ to increase the distance between $z_{v_{i}}$ and the hard negative sample $z_{qa_{j}}$. Therefore, the model can effectively avoid the False Negative Trap caused by hard negative pairs, thereby enhancing the robustness of the inputs $F_{v}$ and $F_{t}$ in scenarios where they are visually similar but clinically distinct.

#### 3.2.3. Feature Fusion via Cross-Modal Attention (FF-CMA)

**Figure 3:**
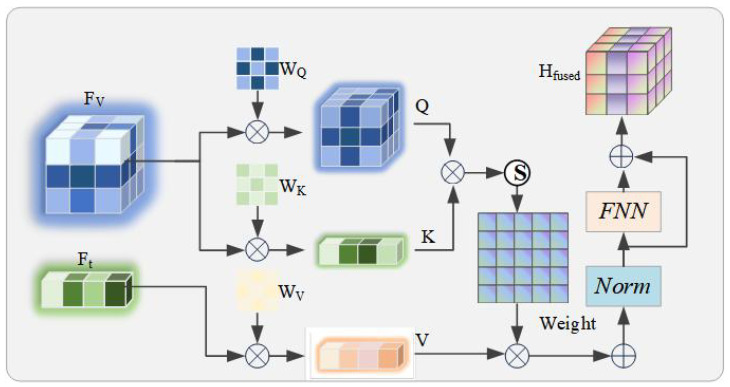
Architecture diagram of the Feature-Fused Cross-Modal Attention (FF-CMA) module. This module is designed to generate image–text fused representations $H_{fused}$.

Based on the enhanced $F_{v}$ and $F_{t}$ from the previous section, which are robust to hard negative samples, we employ a Feature Fusion via Cross-Modal Attention(FF-CMA) to establish the relationship between $F_{v}$ and $F_{t}$. The overall framework of FF-CMA is shown in Figure 3.

This relationship accurately binds an image to its corresponding text and enhances the model’s accuracy in aligning image–text pairs in scenarios that are visually similar yet clinically distinct. Specifically, we use the textual feature sequence $F_{t}$ as the Query, and the visual feature sequence $F_{v}$ as both the Key and the Value. These inputs are first transformed through learnable linear projections:

(6) $$Q=F_{t}W_{Q},\;K=F_{v}W_{K},\;V=F_{v}W_{V},$$

where $W_{Q},\;W_{K},\;W_{V}\in R^{d\times d_{k}}$ are the corresponding projection matrices. The calculation of the attention weights and their output follows the scaled dot-product attention paradigm:

(7) $$\mathrm{Attention}\left(Q,K,V\right)=\mathrm{softmax}\left(\frac{QK^{T}}{\sqrt{d_{k}}}\right)V.$$

To stabilize training and integrate context, we adopt a standard Transformer block structure that includes residual connections and Layer Normalization, followed by a feed-forward network (FFN), to ultimately generate a “text-aware” fused feature representation $H_{\mathrm{fused}}$:

(8) $$H_{\mathrm{fused}}^{\prime }=\mathrm{LayerNorm}\left(\mathrm{Attention}\left(Q,K,V\right)+F_{t}\right)$$

(9) $$H_{\mathrm{fused}}=\mathrm{LayerNorm}\left(\mathrm{FFN}\left(H_{\mathrm{fused}}^{\prime }\right)+H_{\mathrm{fused}}^{\prime }\right).$$

The representation $H_{\mathrm{fused}}$ captures the fine-grained correspondence between the image and text and is fed into the subsequent modules for processing.

### 3.3. Prompt-Based Knowledge Consolidation Module (PKC)

**Figure 4:**
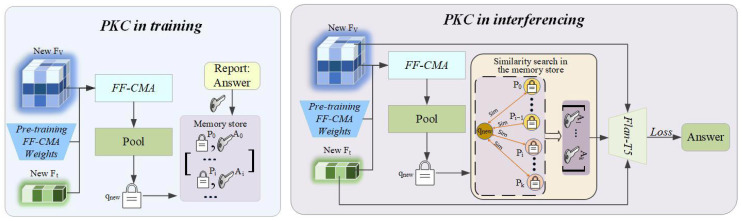
Operational workflow of the Prompt-based Knowledge Consolidation (PKC) module. (**Left**) During training, new knowledge is encoded and incrementally added to a non-parametric memory store in a rehearsal-free manner. (**Right**) During inference, a retrieval-augmented generation process retrieves relevant historical exemplars to provide context for generating fact-based answers.

To address the “stale knowledge” problem of traditional models when encountering new information and to provide a traceable basis for the model’s answers, we introduce the Prompt-based Knowledge Consolidation Module (PKC). It is a framework based on Retrieval-Augmented Generation, whose core advantage lies in decoupling stable, consolidated reasoning capabilities from a dynamic, updatable knowledge base. The overall framework of PKC is shown in Figure 4.

#### 3.3.1. Memory as a Non-Parametric Knowledge Store

The core of the PKC is an external, persistent, and non-parametric memory store M, which stores a vast number of medical knowledge exemplars in the form of key–value pairs. After the CSDM module has completed its training, its parameters $\theta_{\mathrm{CSDM}}$ are permanently frozen. We use this frozen CSDM module (denoted as $\mathrm{CSDM}(\cdot ;\theta_{\mathrm{CSDM}}^{*})$) to process the ROCO-v2 [53] dataset offline and build the initial version of the memory store. For each VQA triplet $\left(V_{i},Q_{i},A_{i}\right)$ in the dataset, we encode it into a single-vector key $p_{i}\in R^{d}$ for efficient retrieval:

(10) $$p_{i}=\mathrm{Pool}\left(\mathrm{CSDM}\left(V_{i},Q_{i};\theta_{\mathrm{CSDM}}^{*}\right)\right),$$

where Pool(·) can be an average pooling operation. This vector key $p_{i}$, along with the corresponding raw answer string $A_{i}$, forms a key–value pair $\left(p_{i},A_{i}\right)$, and is stored in the memory store:

(11) $$M\left[0\right]=\{\left(p_{i},A_{i}\right)|i=1,\ldots ,N_{\mathrm{treatmem}}\},$$

where $N_{\mathrm{treatmem}}$ is the total number of exemplars in the initial memory store. The framework’s knowledge update capability is entirely transferred to the non-parametric memory store M. When a new knowledge dataset $D_{\mathrm{new}}$ becomes available, we use the same frozen CSDM encoder to generate key–value pairs for the new data and add them incrementally to the memory store; this process never modifies any model parameters:

(12) $$M\left[t+1\right]=M\left[t\right]\cup \{\left(\mathrm{Pool}\left(\mathrm{CSDM}\left(V_{j},Q_{j};\theta_{\mathrm{CSDM}}^{*}\right)\right),A_{j}\right)|\left(V_{j},Q_{j},A_{j}\right)\in D_{\mathrm{new}}\}.$$

This design fundamentally solves the “stale knowledge” problem of traditional models, as it allows the knowledge base to stay synchronized with the latest medical advancements without the need for costly and risky full model retraining.

#### 3.3.2. Retrieval-Augmented Knowledge Replay

During the inference phase, when a new query $\left(V_{\mathrm{new}},Q_{\mathrm{new}}\right)$ arrives, the system follows a standard retrieval-augmented-generation workflow. First, the model uses its fixed CSDM module to encode the new query into a query vector $q_{\mathrm{new}}$:

(13) $$q_{\mathrm{new}}=\mathrm{Pool}\left(CSDM\left(V_{\mathrm{new}},Q_{\mathrm{new}};\theta_{CSDM}^{*}\right)\right).$$

Subsequently, the system uses $q_{\mathrm{new}}$ as a query to perform an efficient similarity search in the memory store M to retrieve the *k* most relevant historical exemplars. This retrieval process aims to find a set of indices $J_{k}$ whose corresponding keys have the highest cosine similarity to the query vector:

(14) $$J_{k}=\underset{J\subseteq M,|J|=k}{arg TopK}\sum_{j\in J}\mathrm{sim}\left(q_{\mathrm{new}},p_{j}\right).$$

The retrieved *k* answers $A_{j}|j\in J_{k}$ are concatenated into a single contextual prompt string $C_{\mathrm{retrieved}}=\mathrm{concat}\left(A_{j_{1}},\ldots ,A_{j_{k}}\right)$. Finally, this retrieved context, along with the original question $Q_{\mathrm{new}}$, forms an augmented prompt that is fed into the generative decoder to generate the final answer, guided by the fused features of the current query, $H_{\mathrm{fused},\mathrm{new}}$.

### 3.4. Training and Inference

This section will integrate the aforementioned modules and introduce the model’s overall training objective and inference process.

#### 3.4.1. Training Objective

In the initial training phase (i.e., before the CSDM module weights are frozen), the entire model is guided by a composite loss function. This function is designed to simultaneously shape the model’s generative capabilities and its core debiased discriminative ability. The total loss $L_{\mathrm{total}}$ is a weighted sum of the autoregressive generation loss $L_{\mathrm{LM}}$ and the unified debiased contrastive loss $L_{\mathrm{DCL}}$ defined in Section 3.2.3:

(15) $$L_{\mathrm{total}}=\lambda L_{\mathrm{LM}}+L_{\mathrm{DCL}},$$

where λ is a hyperparameter that balances the contributions of the generation task and the contrastive learning task. $L_{\mathrm{LM}}$ is the core of the model’s generative capability and adopts the standard autoregressive cross-entropy loss. It aims to maximize the probability of generating the ground-truth answer sequence $A=(a_{1},a_{2},\ldots ,a_{T})$, given the fused feature representation $H_{\mathrm{fused}}$:

(16) $$L_{\mathrm{LM}}=-\sum_{t=1}^{T}\log P\left(a_{t}|a_{<t},A_{i}\right),$$

to enhance the model’s image–question–answer matching accuracy, while, through $L_{\mathrm{DCL}}$, improving the model’s robustness to ’confusing samples’, thereby improving the overall performance of multimodal alignment.

#### 3.4.2. Autoregressive Answer Generation

During the inference phase, the answer generation for a novel image–question pair $\left(V_{\mathrm{new}},Q_{\mathrm{new}}\right)$ follows a carefully designed multi-step deterministic process to ensure the accuracy and trustworthiness of the results. The process begins by using the CSDM module with its frozen parameters to encode the input, producing a unified multimodal fused feature representation $H_{\mathrm{fused}}$. This high-dimensional representation encapsulates the fine-grained semantic alignment information between the relevant visual evidence in the image and the question text, serving as a critical bridge connecting perception with subsequent cognitive reasoning. Subsequently, $H_{\mathrm{fused}}$ is used by the Prompt-based Knowledge Consolidation module to perform retrieval-based knowledge enhancement.

Specifically, a query vector is generated by pooling $H_{\mathrm{fused}}$ and is used to perform efficient similarity retrieval in the non-parametric memory store M to locate the *K* most relevant historical medical exemplars. The answer texts corresponding to these exemplars are concatenated into a single contextual string $C_{\mathrm{retrieved}}$, providing external knowledge support for the generation process. Ultimately, the answer generation task is undertaken by the autoregressive decoder of Flan-T5. For its pseudocode, refer to the Appendix A. As the decoder generates each token, its state is not only guided by the augmented text prompt composed of the original question $Q_{\mathrm{new}}$ and the retrieved context $C_{\mathrm{retrieved}}$, but more importantly, it is continuously conditioned on $H_{\mathrm{fused}}$ to integrate visual information via the cross-modal attention mechanism. Regarding the choice of decoding strategy, considering that the determinism and reproducibility of the output are crucial in the high-risk medical domain, this study explicitly and exclusively adopts the Beam Search strategy. This strategy, compared to greedy search, which is prone to getting trapped in local optima, and various sampling methods (such as Top-p sampling) that introduce uncontrollable randomness, can more stably explore and output the answer sequence with the highest overall probability, thereby mechanistically ensuring the reliability and safety of the model’s output results.

## 4. Experiment

This section aims to provide a comprehensive and in-depth empirical evaluation of the Evolvable Clinical-Semantic Alignment (ECSA) framework proposed in this paper through a series of rigorous experiments. Our experimental design revolves around three core objectives: first, to compare ECSA with SOTA models across multiple dimensions on standard in-domain evaluation benchmarks; second, to quantify the generalization capability of ECSA in few-shot scenarios through specially designed dataset adaptation experiments and directly compare it with key related works; and finally, to dissect the independent contributions and synergistic effects of each key component within the framework through exhaustive ablation studies.

### 4.1. Datasets

To comprehensively evaluate the model performance and construct the knowledge enhancement module, this study utilized a total of five public datasets. VQA-RAD, SLAKE, PathVQA, and VQA-Med-2019 were used for model training and evaluation, while ROCO-v2 [53] was specifically used as an external knowledge base.

VQA-RAD: This is a radiology VQA dataset manually constructed by clinicians. The dataset contains 315 unique radiology images (such as CT, MRI, X-rays), covering multiple body parts including the head, chest, and abdomen, and is accompanied by 3515 question–answer pairs.

SLAKE: This is a larger and more diverse bilingual (English and Chinese) Med-VQA dataset. Following common practice, we only use its English portion, which contains 642 images and approximately 7000 question–answer pairs. Compared to VQA-RAD, SLAKE covers a broader range of imaging modalities and anatomical regions. One of its notable features is that, in addition to questions based purely on visual content, it also includes knowledge-based questions that require external medical knowledge to answer.

PathVQA: This is a VQA dataset focused on pathology images, containing 4998 images and nearly 33,000 question–answer pairs extracted from pathology textbooks and digital libraries. A key feature of this dataset is the high proportion of open-ended questions (approximately 50.2%), which presents a greater challenge for the models.

VQA-Med-2019: As part of the ImageCLEF challenge, this dataset focuses on radiology images, with a training set of 3200 images and 12,792 question–answer pairs. Its questions are primarily centered around four categories: Modality, Plane, Organ System, and Abnormality, designed to test the model’s ability to answer based solely on image content.

ROCO-v2 (Radiology Objects in COntext version 2): This is a large-scale radiology multimodal dataset containing over 81,000 images extracted from public PubMed Central articles and their corresponding captions. The dataset covers multiple imaging modalities such as CT, MRI, and X-ray.

### 4.2. Evaluation Metrics

To comprehensively evaluate the model performance from different dimensions, we adopted two core evaluation metrics. Accuracy is the primary performance basis in all experimental scenarios. Additionally, to specifically quantify the model’s knowledge retention capability in the ablation studies (Section 4.6), we also introduced the Forgetting rate (F).

Accuracy: In line with the prevailing evaluation practices in the Med-VQA community, we perform exact-string matching to determine correctness. This metric serves as the primary yardstick for model performance, and we formulate it via the confusion-matrix-based definition:

(17) $$\mathrm{Accuracy}=\frac{\mathrm{TP}+\mathrm{TN}}{\mathrm{TP}+\mathrm{TN}+\mathrm{FP}+\mathrm{FN}},$$

where TP, TN, FP, and FN denote true positives, true negatives, false positives, and false negatives, respectively, obtained by treating each unique textual answer as a distinct class. We report accuracy on the overall, open-ended, and closed-ended question subsets.

Forgetting (F): This metric is specifically used in the ablation studies to quantify the model’s knowledge retention capability during the continual learning process. It measures the degree to which the model’s performance on old tasks degrades after learning a new task. Its calculation formula is as follows:

(18) $$F_{K}=\frac{1}{K-1}\sum_{j=1}^{K-1}\left(a_{j,j}-a_{K,j}\right),$$

where $a_{K,j}$ represents the accuracy of the model on the test set of the *j*-th task after having learned the *K*-th task, and $a_{j,j}$ represents the best accuracy achieved by the model right after learning the *j*-th task. The lower the value of the forgetting rate *F*, the milder the catastrophic forgetting phenomenon.

### 4.3. Experimental Setup

#### 4.3.1. Implementation Details

Our ECSA framework is based on two powerful pre-trained foundation models. The visual encoder uses the ViT-B/16 model from BiomedCLIP (approx. 86 million parameters), and the language model is Flan-T5-Base, which contains approximately 250 million parameters. Crucially, our parameter-efficient approach freezes the backbones of these large models. The trainable parameters are limited only to the Clinical-Semantic Disambiguation module, as the Prompt-based Knowledge Consolidation module is a non-parametric memory store with no trainable weights. This trainable module totals approximately 8.4 million parameters, representing less than 3% of the entire model’s parameter count. All models are trained with a unified hyperparameter setup: we use the AdamW optimizer with an initial learning rate set to $1\times 10^{-4}$ and employ a linear decay learning rate scheduling strategy. The batch size is set to 16. In our composite loss function, the weight hyperparameter λ that balances the generation loss and the contrastive loss is set to 1.0. In the CSDM module, the temperature hyperparameter τ for the InfoNCE loss is set to 0.07, and the similarity threshold θ for debiased hard negative mining is set to 0.85. During the inference phase, the Prompt-based Knowledge Consolidation module retrieves the k=3 most relevant exemplars. All experiments are conducted on a single NVIDIA GeForce RTX 3090 GPU with 24 GB of VRAM.

#### 4.3.2. Hyperparameter Sensitivity Analysis

**Figure 5:**
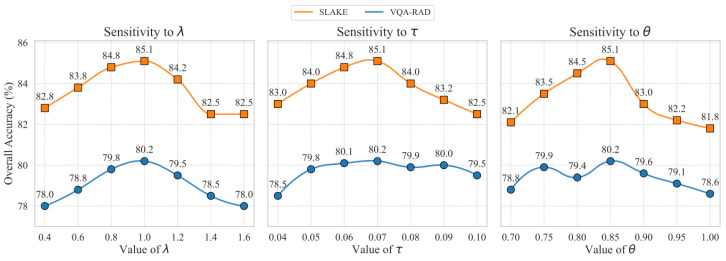
Sensitivity analysis for key hyperparameters on the VQA-RAD and SLAKE validation sets. (**Left**) Model accuracy across different values of the loss weight λ. (**Center**) Model accuracy as a function of the contrastive loss temperature τ. (**Right**) Model accuracy under varying similarity thresholds θ for the DHNM strategy. Performance peaks indicate the empirically determined optimal values.

We conducted a sensitivity analysis on the selection of key hyperparameters to provide an experimental basis for their values, with the experimental data shown in Figure 5. To determine the optimal configuration, we adopted a controlled experiment approach: when validating a specific hyperparameter, all other parameters were kept at the default values described in Section 4.3.1. The results in Figure 5 provide a solid empirical basis for our hyperparameter choices. The experiments were conducted on the validation sets of VQA-RAD and SLAKE, respectively. The results show that when the loss function weight λ=1.0, the model achieves the best balance between the generation task and the contrastive learning task. For the contrastive loss temperature τ, a setting of 0.07 best balances the focus on hard negative samples. For the hard negative mining threshold θ, a threshold of 0.85 strikes the optimal balance between effectively filtering semantically similar “false negative” samples and retaining a sufficient number of hard negative samples, thereby maximizing the model’s discriminative ability.

### 4.4. Comparison with SOTA Models

To place the performance of ECSA in the broader context of Med-VQA research, this section compares its performance under the standard in-domain evaluation paradigm with several non-continual learning SOTA models that have reported the best performance on the VQA-RAD, SLAKE, PathVQA, and VQA-Med-2019 test sets. All comparisons use publicly verifiable data reported in the original papers of the baseline models, ensuring the fairness and transparency of the comparison.

The data in Table 1 contextualize the performance of ECSA across four distinct datasets, benchmarking it against current SOTA models. On the two primary radiology datasets, VQA-RAD and SLAKE, ECSA achieves highly competitive overall accuracies of 80.15% and 85.10% respectively, outperforming several recent models like LaPA and performing on par with AMiF. Notably, on VQA-RAD’s more challenging open-ended questions, ECSA’s accuracy of 71.25% is particularly outstanding.

To further assess the model’s robustness, its performance was evaluated on two additional datasets. On VQA-Med-2019, another radiology dataset, the ECSA achieves a strong accuracy of 82.23%, demonstrating excellent in-domain generalization. On the more challenging cross-domain PathVQA dataset, which focuses on pathology and features a higher proportion of difficult open-ended questions, the ECSA attains an overall accuracy of 64.57%. While the demanding nature of its open-ended questions (39.64%) impacts the overall score, the model maintains a high accuracy of 89.50% on closed-ended questions, confirming the solidity of its foundational capabilities.  

This comprehensive performance profile strongly supports our framework’s design philosophy. Models like AMiF typically adopt an end-to-end fine-tuning strategy to achieve peak performance on a single fixed dataset. In contrast, ECSA’s parameter-efficient approach—freezing most of the backbone network—achieves comparable or superior performance on multiple radiology benchmarks while also demonstrating reasonable generalization to a new medical domain. This validates that our design achieves an excellent balance between high performance and an evolvable efficient architecture.

### 4.5. Generalization Ability Evaluation

To evaluate the model’s generalization capability in few-shot scenarios, we follow the dataset adaptation (DA) paradigm from the paper “Multimodal Prompt Retrieval for Generative Visual Question Answering” (MPR) and directly compare with its reported results. The experimental setup is a k = 1 few-shot domain adaptation, where after the model is trained on a source dataset, during inference on the target dataset, it is allowed to retrieve one (k = 1) most similar question–answer pair from the target dataset as a contextual prompt.

The results in Table 2 clearly indicate that ECSA demonstrates a significant performance advantage in the few-shot domain adaptation task, comprehensively outperforming the MPR model, which serves as the basis for our work. In the adaptation task from SLAKE to VQA-RAD, ECSA achieves an overall accuracy of 67.5%, a significant improvement of 4.5 percentage points over MPR’s 63.0%. In the reverse adaptation task from VQA-RAD to SLAKE, ECSA also substantially leads MPR with an overall accuracy of 58.2%. This result is significant as it directly validates the effectiveness of our framework’s design. The performance improvement of ECSA is primarily attributed to its superior retrieval capability, which is driven by the Prompt-based Knowledge Consolidation Module (PKC). As an efficient Retrieval-Augmented Generation (RAG) mechanism, PKC can accurately find the most relevant historical exemplars for the current query in few-shot scenarios. This superior retrieval capability is made possible because the query vectors and memory store keys it relies on for retrieval are both generated by the Clinical-Semantic Disambiguation Module (CSDM). Through its hierarchical alignment and debiased hard negative mining strategy, CSDM can produce more discriminative image–text joint embeddings. Therefore, it is the synergistic effect between the high-quality representations provided by CSDM and the efficient retrieval mechanism of PKC that enables ECSA to achieve superior performance in few-shot generalization tasks.

### 4.6. Ablation Study

To thoroughly validate the actual contributions of each innovative component in the ECSA framework, we conducted a series of rigorous ablation studies. By systematically removing or replacing key modules within the framework, we can quantify the impact of each part on the model’s final performance.

Our study begins with the validation of the base model selection. Before constructing a complex framework, it is essential to first establish a solid foundation. Therefore, starting with the generic models used in MPR (Generic CLIP + Generic T5), we progressively replace them with domain-specific models through ablation studies to demonstrate the rationale for our choices.

The results in Table 3 clearly demonstrate the importance of domain-specific models. Starting from the generic model combination, whether individually replacing the visual encoder with the biomedically pre-trained BiomedCLIP or individually replacing the language model with the instruction-finetuned Flan-T5, both lead to performance improvements on both datasets. This, respectively, highlights the importance of a domain-specific visual encoder for understanding subtle pathological features in medical images and the advantage of an instruction-finetuned language model for accurately parsing and generating complex medical question–answers. When we use both BiomedCLIP and Flan-T5 simultaneously, the performance is optimal, proving that their combination provides the most powerful foundation for our framework.

After establishing the optimal base model combination, we further analyzed the necessity of several core innovative modules in ECSA. To rigorously evaluate the performance of each component in a continual learning setting, we constructed a challenging sequence of five tasks. This sequence was formed by strategically partitioning the VQA-RAD and SLAKE datasets, aiming to simulate a real-world scenario of Domain-Incremental Learning. The task sequence is as follows: Task 1: radiological abnormality recognition (the ‘abnormality’ category of VQA-RAD), Task 2: multimodal organ localization (the ‘organ system’ category of SLAKE), Task 3: anatomical plane and modality classification (the ‘plane’ and ‘modality’ categories of VQA-RAD), Task 4: general visual attribute question answering (the ‘color’, ‘shape’, etc., categories of SLAKE), and Task 5: comprehensive mixed-domain evaluation (reserved samples from both datasets). This design ensures significant distribution shifts between tasks, providing a stringent testbed for evaluating catastrophic forgetting.

The comprehensive forgetting rate reported in the table is the final value calculated according to the formula $F_{5}=\frac{1}{4}\sum_{j=1}^{4}\left(a_{j,j}-a_{5,j}\right)$ after the model has completed training on all five tasks. This value explicitly quantifies the average performance degradation after the model has learned the entire sequence, relative to the best performance it achieved on each of tasks 1 to 4.

The quantitative results in Table 4 systematically deconstruct the success factors of ECSA. All experiments were conducted on the aforementioned five-task sequence, and the table reports the model’s average accuracy on the two datasets after the completion of sequential learning, as well as the comprehensive forgetting rate over the entire sequence. The results clearly demonstrate the contribution of each component in the framework to maintaining knowledge stability. The full ECSA model achieved a comprehensive forgetting rate of 18.5%. Although this design is not intended to compete with the current state-of-the-art continual learning methods that rely on data replay, the effectiveness of its internal components has been fully validated. When the Prompt-based Knowledge Consolidation module (w/o PKC), which serves as the core memory mechanism, was removed, the model’s performance experienced a catastrophic collapse, with the forgetting rate soaring to 33.2%. This irrefutably establishes that the PKC is the cornerstone for mitigating catastrophic forgetting within the framework. Similarly, when the Clinical-Semantic Disambiguation module (w/o CSDM) was removed, the forgetting rate also rose significantly to 26.1%, which indicates that the high-quality aligned representations produced by the CSDM module are crucial for the effective accumulation and consolidation of subsequent knowledge. Finally, after replacing our proposed debiased hard negative mining strategy with standard negative sampling (w/o Debiased Mining), the forgetting rate also increased to 21.3%. This clearly shows that our proposed mining strategy not only enhances the model’s discriminative ability, but the more robust and refined medical representations it learns are also more conducive to resisting knowledge forgetting.

### 4.7. Qualitative Analysis

**Figure 6:**
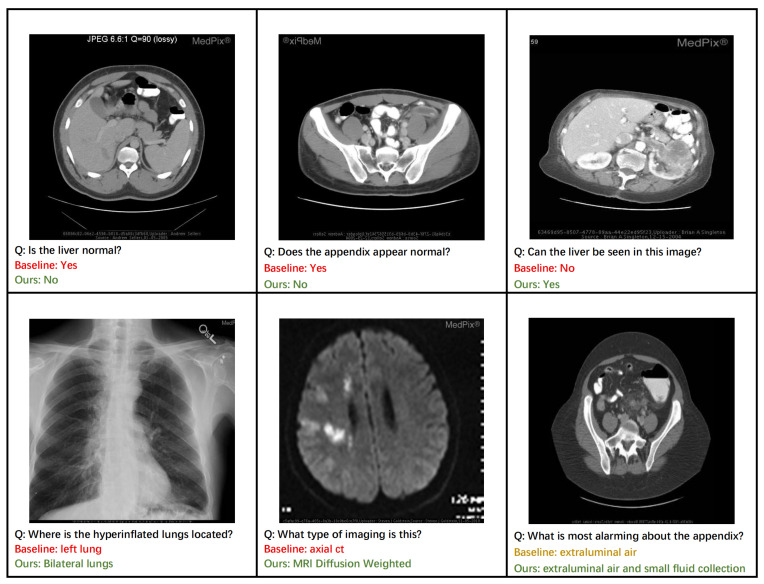
Qualitative comparison between ECSA and a baseline model on representative clinical cases. (**Top Row**) ECSA demonstrates superior diagnostic accuracy, correctly identifying abnormalities in the liver and appendix that were missed by the baseline. (**Bottom Row**) ECSA generates more comprehensive and clinically precise answers, such as identifying bilateral pathology and specific imaging modalities.

Figure 6 provides a qualitative analysis that highlights the practical advantages of our ECSA framework. Our model demonstrates superior diagnostic accuracy, correctly identifying abnormalities in the liver and appendix, where a baseline approach might fail (top row). This enhanced discriminative capability is a direct result of the Clinical-Semantic Disambiguation Module (CSDM). By employing a debiased hard negative mining strategy, CSDM learns highly robust representations that can distinguish subtle pathological markers from normal tissue, which is crucial for accurate initial assessments.

Furthermore, the ECSA exhibits a capacity for more comprehensive and clinically nuanced reasoning. For instance, it correctly identifies pathology as affecting ‘bilateral lungs’ rather than a single lung (bottom row, left) and provides a more complete description of findings related to the appendix, noting both ‘extraluminal air and small fluid collection’ (bottom row, right). This ability to generate detailed and complete answers stems from the rich context-aware features produced by CSDM’s cross-modal attention mechanism. The model’s proficiency in knowledge-intensive tasks is also evident, as it correctly identifies the specific imaging modality as ‘MRI Diffusion Weighted’ (bottom row, middle). This level of specificity is enabled by the Prompt-based Knowledge Consolidation Module (PKC), which functions as a retrieval-augmented knowledge base to supply relevant factual information, grounding the model’s response in a vast repository of clinical examples. Together, these cases illustrate how ECSA’s components synergistically contribute to generating responses that are not only more accurate but also more clinically precise and reliable.

## 5. Conclusions

This paper addresses the two long-standing core challenges in the field of Medical Visual Question Answering—weak few-shot generalization and catastrophic forgetting in continual learning—by proposing an innovative unified framework named the Evolvable Clinical-Semantic Alignment framework. This framework achieves efficient knowledge alignment, transfer, and accumulation in a parameter-efficient manner, built upon frozen pre-trained large vision and language models.

Our main contributions are embodied in two meticulously designed modules:

1. Clinical-Semantic Disambiguation Module: By introducing a novel debiased hard negative mining strategy, it effectively addresses the “False Negative Trap” of traditional contrastive learning in medical scenarios, significantly enhancing the model’s ability to discriminate difficult cases that are visually similar but clinically distinct;
2. Prompt-based Knowledge Consolidation Module: Acting as a rehearsal-free dynamic knowledge base, it successfully retains knowledge learned from old tasks without storing any past patient data, thereby effectively overcoming catastrophic forgetting;

Comprehensive experimental evaluations quantitatively validate the effectiveness of our framework. ECSA achieves state-of-the-art or highly competitive performance across four public benchmarks, attaining overall accuracies of 80.15% on VQA-RAD and 85.10% on SLAKE, while maintaining this performance by fine-tuning less than 3% of the total parameters. In a challenging one-shot cross-dataset generalization task, the framework demonstrates a significant 4.5 percentage point improvement over strong baselines, directly validating the synergy between CSDM’s discriminative representations and PKC’s retrieval capabilities. Critically, in a five-task continual learning scenario, ECSA achieves a low forgetting rate of just 13.50%. Ablation studies confirm that this robust knowledge retention is primarily driven by the PKC module, the removal of which causes the forgetting rate to increase to 32.20%.

Limitations: While the ECSA has demonstrated promising results, we acknowledge several limitations that open avenues for future exploration. First, the scalability and generalizability of our framework were tested on standard public benchmarks, which, while representative, are limited in size and diversity compared to real-world clinical data. The model’s performance on larger institution-specific datasets remains to be validated. Second, while the framework was tested across radiology and pathology, its adaptability to other imaging modalities requires further investigation.

Future Work: Based on these limitations, future work could proceed in two main directions: (1) Conducting large-scale clinical validation. Applying the framework to broader institution-specific datasets and more diverse imaging modalities is key to verifying its ultimate value in real-world clinical workflows. (2) Integrating structured knowledge. Future research could explore integrating medical knowledge graphs into the PKC module to enhance the model’s complex reasoning capabilities. In conclusion, this research provides a promising path for building more robust, adaptive, and trustworthy next-generation clinical intelligent assistant systems.

## Figures and Tables

**Table 1:** Accuracy of models on the VQA-RAD, SLAKE, PathVQA, and VQA-Med-2019 test sets. In the table, bold indicates the best result, and underlined values denote the second-best.

Methon	Year	VQA-RAD(%)			SLAKE(%)			PathVQA(%)			VQA-2019(%)
		Overall	Open	Closed	Overall	Open	Closed	Overall	Open	Closed	Overall
**PubMedCLIP [54]**	2021	72.10	60.10	80.00	80.10	78.40	82.50	-	-	-	-
**M2I2 [55]**	2022	76.80	66.50	83.50	81.20	74.70	**91.10**	62.20	36.30	88.00	-
**BiomedCLIP [51]**	2023	75.20	67.60	79.80	85.40	82.50	89.70	-	-	-	-
**PMC-VQA [56]**	2023	-	**73.70**	86.80	-	84.50	86.30	-	-	-	61.00
**MPR [16]**	2023	73.20	62.60	80.10	79.40	77.50	82.20	59.63	36.03	82.47	75.93
**MUMC [57]**	2023	79.29	71.50	84.20	84.90	-	-	65.10	39.00	90.40	-
**PeFoMed [58]**	2024	77.40	62.60	87.10	82.10	77.80	88.70	63.60	35.70	**91.30**	-
**LaPA [59]**	2024	79.38	68.72	86.40	84.73	82.17	86.40	-	-	-	81.60
**AMiF [47]**	2025	**80.49**	72.00	**87.25**	**86.71**	**84.84**	90.23	**65.33**	**40.33**	90.18	**83.20**
**PLAF (Ours)**	-	80.15	71.25	86.85	85.10	82.90	89.30	64.57	39.64	89.50	82.23

**Table 2:** Cross-dataset evaluation results.

Source → Target	Method	Open	Closed	Overall
SLAKE → VQA-RAD	MPR	42.9%	76.2%	63.0%
	ECSA (Ours)	48.5%	79.8%	67.5%
VQA-RAD → SLAKE	MPR	45.1%	67.3%	53.8%
	ECSA (Ours)	49.5%	71.5%	58.2%

**Table 3:** Results of ablation study on base model selection.

Base Model Configuration	VQA-RAD Overall	SLAKE Overall
Generic CLIP + Generic T5 (MPR Base)	73.2%	79.4%
BiomedCLIP + Generic T5	74.5%	81.8%
Generic CLIP + Flan-T5	74.2%	81.5%
BiomedCLIP + Flan-T5 (Ours)	75.0%	82.0%

**Table 4:** Results of ablation study on key components of ECSA.

Model Variant	VQA-RAD Average Accuracy	SLAKE Average Accuracy	Forgetting
ECSA	80.15%	85.10%	13.50%
w/o PKC	55.10%	60.30%	32.20%
w/o CSDM	68.90%	73.10%	21.10%
w/o Debiased Mining	71.50%	75.80%	19.30%

## Data Availability

The datasets used and analyzed in this study are all publicly available.

## Appendix A

**Algorithm A1** BiomedCLIP Vision Transformer Encoder
**Input:** Image $I\in R^{H\times W\times 3}$ **Output:** Sequence of visual features $F_{v}\in R^{N_{v}\times d_{v}}$ 1: **function** ViT_Encoder(*I*) 2: $X_{p}\leftarrow \mathrm{ImageToPatches}\left(I,P\right)$ 3: $X_{flat}\leftarrow \mathrm{Flatten}\left(X_{p}\right)$ 4: $E\leftarrow \mathrm{LinearProjection}(X_{flat})$ 5: $x_{class}\leftarrow \mathrm{LearnableClassToken}\left(\right)$ 6: $Z_{0}\leftarrow \mathrm{Concat}\left(\left[x_{class},E\right]\right)$ 7: $Z_{0}\leftarrow Z_{0}+\mathrm{PositionalEncoding}$ 8: **for** l=1 **to** *L* **do** 9: $A_{l}\leftarrow \mathrm{LayerNorm}\left(Z_{l-1}\right)$ 10: $A_{l}\leftarrow \mathrm{MultiHeadSelfAttention}\left(Q=A_{l},K=A_{l},V=A_{l}\right)$ 11: $Z_{l}^{\prime }\leftarrow Z_{l-1}+A_{l}$ 12: $F_{l}\leftarrow \mathrm{LayerNorm}\left(Z_{l}^{\prime }\right)$ 13: $F_{l}\leftarrow \mathrm{FeedForwardNetwork}\left(F_{l}\right)$ 14: $Z_{l}\leftarrow Z_{l}^{\prime }+F_{l}$ 15: **end for** 16: $F_{v}\leftarrow \mathrm{LayerNorm}\left(Z_{L}\right)$ 17: **return** $F_{v}$ 18: **end function**

**Algorithm A2** Flan-T5 Textual Context Encoder
**Input:** Instruction $I_{task}$, Question *Q*, Ground-truth Answer *A* **Output:** Textual features $F_{t}\in R^{N_{t}\times d_{t}}$ 1: **function** TextualContextEncoder($I_{task},Q,A$) 2: $T_{combined}\leftarrow \mathrm{Concatenate}\left(I_{task},Q,A\right)$ 3: $E_{0}\leftarrow \mathrm{TokenEmbedding}\left(T_{combined}\right)+\mathrm{PositionalEncoding}$ 4: **for** l=1 **to** $L_{enc}$ **do** 5: $E_{l}\leftarrow \mathrm{TransformerEncoderLayer}\left(E_{l-1}\right)$ 6: **end for** 7: $F_{t}\leftarrow \mathrm{LayerNorm}\left(E_{l}\right)$ 8: **return** $F_{t}$ 9: **end function**

**Algorithm A3** Flan-T5 Autoregressive Answer Generation (Inference)
**Input:** Fused multimodal features $H_{fused}$, New question $Q_{new}$, Memory store M **Output:** Generated answer sequence $A_{gen}$ **function** AnswerGeneration($H_{fused},Q_{new},M$) $q_{retrieval}\leftarrow \mathrm{Pool}\left(H_{fused}\right)$ $C_{retrieved}\leftarrow \mathrm{RetrieveExemplars}\left(q_{retrieval},M,K\right)$ $P_{prompt}\leftarrow \mathrm{Concatenate}\left(Q_{new},C_{retrieved}\right)$ $D_{0}\leftarrow \mathrm{TokenEmbedding}\left(P_{prompt}\right)+\mathrm{PositionalEncoding}$ $A_{gen}\leftarrow \mathrm{BeamSearchInitialize}\left(D_{0}\right)$ **for** i=1 **to** MaxLength **do** $D_{in}\leftarrow \mathrm{GetLastHiddenStates}\left(A_{gen}\right)$ **for** l=1 **to** $L_{dec}$ **do** $D_{l}^{\prime }\leftarrow \mathrm{MaskedMultiHeadAttention}\left(Q=D_{in},K=D_{in},V=D_{in}\right)$ $D_{l}^{\prime }\leftarrow \mathrm{LayerNorm}\left(D_{in}+D_{l}^{\prime }\right)$ $D_{l}^{\prime \prime }\leftarrow \mathrm{MultiHeadAttention}\left(Q=D_{l}^{\prime },K=H_{fused},V=H_{fused}\right)$ $D_{l}^{\prime \prime }\leftarrow \mathrm{LayerNorm}\left(D_{l}^{\prime }+D_{l}^{\prime \prime }\right)$ $D_{out}\leftarrow \mathrm{FeedForwardNetwork}\left(D_{l}^{\prime \prime }\right)$ $D_{in}\leftarrow \mathrm{LayerNorm}\left(D_{l}^{\prime \prime }+D_{out}\right)$ **end for** $A_{gen}\leftarrow \mathrm{BeamSearchStep}\left(A_{gen},D_{in}\right)$ **end for** **return** $\mathrm{BestHypothesis}(A_{gen})$ **end function**
